# Implementing the 2013 WHO diagnostic criteria for gestational diabetes mellitus in a Rural Nigerian Population

**DOI:** 10.11604/pamj.2020.36.208.20818

**Published:** 2020-07-22

**Authors:** Ayokunle Moses Olumodeji, Raymond Akujuobi Okere, Idowu Oluwaseyi Adebara, Gbadebo Oladimeji Ajani, Olumide Emmanuel Adewara, Segun Murtala Ghazali, Ufuoma Oluwaseyi Olumodeji

**Affiliations:** 1 Institute of Maternal and Child Health, Lagos State University Teaching Hospital, Lagos, Nigeria,; 2 Department of Obstetrics and Gynaecology, Federal Teaching Hospital, Ido-Ekiti, Ekiti State, Nigeria,; 3 Endocrinology Unit, Department of Medicine, Federal Teaching Hospital, Ido-Ekiti and Afe Babalola University Ado-Ekiti, Ekiti State, Nigeria,; 4 Department of Chemical Pathology, Federal Teaching Hospital, Ido-Ekiti, Ekiti State, Nigeria,; 5 Department of Ophthalmology, Lagos State University Teaching Hospital, Lagos, Nigeria

**Keywords:** Gestational diabetes mellitus, pregnancy, WHO GDM diagnostic criteria, diabetes in pregnancy

## Abstract

**Introduction:**

the World Health Organization (WHO) reviewed the threshold values required for the diagnosis of Gestational Diabetes Mellitus (GDM) in 2013 and the implementation of the new diagnostic criteria have been associated with increase in the prevalence of GDM in some populations. The new cohort of pregnant women that will be labeled to have GDM by the 2013 WHO diagnostic criteria but not by the 1999 WHO diagnostic criteria will pose additional burden to specialized antenatal care, though their pregnancy outcome may not warrant such care. It is thus important to first determine the effect of the implementation of these new consensus diagnostic criteria on the prevalence of GDM in our environment.

**Methods:**

this is a prospective hospital-based study that compared the implementation of both 1999 and 2013 WHO GDM diagnostic criteria among 117 pregnant women who were initially screened with 50-gram Glucose Challenge Test (50-g GCT). Women with a positive Glucose Challenge Test (GCT) result underwent a 75-gram Oral Glucose Tolerance Test (75-g OGTT), which was used as the actual diagnostic test for GDM using both 2013 WHO and 1999 WHO diagnostic criteria. Associations between variables were tested using Chi-square, Fisher's exact and t-test as appropriate. Significance level was set at P value < 0.05.

**Results:**

the prevalence rates of GDM in the study were 2.6% and 7.7% for 1999 WHO and 2013 WHO criteria respectively. Clinical characteristics were similar in women with GDM and women without GDM. The fasting component of the OGTT identified all the women with GDM.

**Conclusion:**

the implementation of the 2013 WHO diagnostic criteria is associated with a 2.5 to 3-fold rise in the prevalence of GDM. Selective risk-factor based screening may be clinically irrelevant with the adoption of the 2013 WHO diagnostic criteria. A minimum of fasting plasma glucose in resource poor settings can be considered to identify women with GDM since it appeared to have 100% sensitivity in our study.

## Introduction

Gestational diabetes mellitus was previously defined as carbohydrate intolerance of variable severity with onset or first recognition during pregnancy [1]. However, WHO in 2013 proposed new criteria for the diagnosis and definition of hyperglycemia first diagnosed in pregnancy that defines gestational diabetes mellitus as diabetes first detected during pregnancy that is not clearly overt diabetes [2].

The global prevalence of diabetes mellitus continues to escalate affecting about 350 million individuals worldwide [3]. Increasing prevalence of overweight and obesity in both developed and developing countries are the main factors for this alarming rise in the diabetic epidemic [4]. Consequently, the prevalence of diabetes among women of child-bearing age is also expected to rise [5].

The 1999 version of World Health Organization´s criteria has been widely used in Nigeria [6]. However, in 2013, WHO revised its recommendations for classifying hyperglycaemia taking into cognizance the issues raised by the International Association of Diabetes in Pregnancy Study Groups (IADPSG) recommendations [2]. In some Caucasian population the implementation of the 2013 WHO diagnostic criteria has been associated with an increase in the prevalence of GDM [7]. The main reason that cases of gestational diabetes have trebled using IADPSG and 2013 WHO criteria is the reliance on a single raised blood sugar result for diagnosis [8].

In developing countries, the implications of applying the 2013 WHO modifications on the prevalence of GDM, the management of hyperglycaemia and the resulting pregnancy outcome is not known [9]. While these recent guidelines aimed to provide a more evidence-based consensus to GDM screening, there remain some concerns about their impact on services, including whether the additional women identified using this approach include a number of women with ‘mild GDM’ whose pregnancy outcomes might not warrant the additional burden of this approach to screening [10]. It is therefore important to determine the proportions of women with GDM diagnosed using 1999 and 2013 WHO diagnostic criteria in a rural population in Nigeria.

## Methods

The Ethics and Research Committee of the Federal Teaching Hospital, Ido-Ekiti, Ekiti State, Nigeria, approved the study protocol (Protocol Number: ERC/2015/11/25/52A). A structured proforma was used to obtain relevant data from each patient. Data obtained from the study participants included age, religion, educational status, occupation, parity, gestational age and history of GDM in previous pregnancies, previous history of macrosomic baby, history of recurrent miscarriages, pre-pregnancy or booking weight, history of diabetes in first degree relative, previous baby with congenital abnormality and previous unexplained still birth. Their height, weight and blood pressure were measured. A load of 50 grams of glucose in 250 mls of water was given to each participant to drink within 5minutes from time zero, without prior dietary restriction, at any time of the day, regardless of whether or not they were fasting and 2 mls of venous blood sample was obtained aseptically from a prominent vein on their forearm into a fluoride oxalate specimen bottle 1 hour from the noted time zero. Plasma glucose level of the blood samples obtained was determined by the glucose oxidase enzyme system [11]. Patients were labelled as screened positive for plasma glucose levels ≥ 140 mg/dl (7.8mmol/l) and screened negative when < 140 mg/dl. After one week all patients screened positive had 75-g OGTT.

The 75-g OGTT test was performed in the morning after 8-14 hours overnight fast. A 5-10 minutes rest period was ensured before commencement of the test in a comfortable waiting area provided for the duration of the test. The study participants were instructed to avoid exercise during the procedure. Blood samples were collected in fluoride oxalate bottles. A blood sample was collected for measurement of fasting glucose before the test was undertaken. A glucose load of 75 grams anhydrous glucose was given orally in a total fluid volume of 250-300mL. The glucose drink was consumed over a 5 minute period. Timing for the rest of the test commenced at the beginning of ingestion and further blood samples were collected at one and two hours from the commencement of the glucose load and the plasma glucose concentrations were measured. The test (other than the fasting sample) was invalid if the patient vomited during the procedure and such patients were rescheduled to repeat the test within the next one week. Plasma glucose estimation of all the taken blood samples was determined using the glucose oxidase enzyme system using Randox kits (Randox Laboratories Limited, UK). Glucose tolerance status was determined based on the 1999 and 2013 diagnostic criteria for 75-g OGTT by WHO [2].

For the 2013 WHO diagnostic criteria, [9] diagnosis of GDM was made using 75-g OGTT when one or more of the following results are recorded: Fasting plasma glucose ≥ 5.1-6.9mmol/L; 1-hour post 75-g oral glucose load ≥ 10mmol/L; 2-hour post 75-g oral glucose load ≥ 8.5-11.0mmol/L. For the 1999 WHO diagnostic criteria [9], diagnosis of GDM was made using 75-g OGTT when one or more of the following results are recorded: Fasting plasma glucose ≥ 7.0 mmol/L; 2-hour post 75-g oral glucose load ≥ 7.8mmol/L.

The data and information obtained from the study participants were processed using statistical package for social sciences version 20 (SPSS Inc., Chicago, Illinois, USA). Frequency tables were generated and the results tested for statistical significance using chi-square and student t-test where appropriate. The level of statistical significance was set at p value < 0.05 at 95% Confidence Interval.

## Results

This study showed no significant difference, in age, parity, previous unexplained intrauterine fetal death, previous delivery of macrosomic babies, previous miscarriages, family history of diabetes mellitus and body mass index, between women who developed GDM and those who did not using the 2013 WHO GDM diagnostic criteria (Table 1).

**Table 1:** clinical characteristics of GDM and non-GDM women

Variables	Non-GDM	GDM	χ2	P value
	N = 108 (%)	n = 9 (%)		
**Age group in yrs, n (%)**				
< 25	2 (1.9)	0 (0.0)		0.925^*^
25-29	39 (36.1)	3 (33.3)		
30-34	32 (29.6)	3 (33.3)		
35+	35 (32.4)	3 (33.4)		
Mean ± SD	31.4 **±** 4.2	33.1 **±** 5.1	1.188	0.237^**^
**Parity**				
Nulliparous	34 (31.5)	1 (11.1)		0.187^*^
Multiparous	74 (68.5)	8 (88.9)		
**Previous unexplained IUFD**				
Yes	9 (8.3)	1 (11.1)		0.566^*^
No	99 (91.7)	8 (88.9)		
**Previous Macrosomia**				
Yes	11 (10.2)	2 (22.2)		0.262^*^
No	97 (89.8)	7 (77.8)		
**Spontaneous Miscarriage**				
Yes	27 (25.0)	3 (33.3)		0.418^*^
No	81 (75.0)	6 (66.7)		
**Family History of Diabetes**				
Yes	11 (10.2)	1 (11.1)		0.636^*^
No	97 (89.8)	8 (88.9)		
**Body Mass Index**				
Normal	31 (28.7)	1 (11.1)		0.292^*^
Overweight	46 (42.6)	6 (66.7)		
Class I Obesity	14 (13.0)	2 (22.2)		
Class II Obesity	17 (15.7)	0 (0.0)		

N- Total number of women without GDM using the 2013 WHO diagnostic criteria n- Total number of women with GDM using the 2013 WHO diagnostic criteria χ^2^ - chi square ^*^Fisher´s exact test applied ^**^ Independent Samples t-test applied IUFD-Intrauterine Fetal Death

The mean plasma glucose values following the 50-g Glucose Challenge Test was significantly higher in women who developed GDM (8.2 ± 1.0 mmol/L) than in women without GDM (5.8 ± 1.2 mmol/L) (p < 0.001) (Table 2). Mean weight and blood pressure were similar in both groups (Table 2).

**Table 2:** comparison of mean values of clinical parameters of women with GDM and women without GDM

	Non-GDM (N = 108)	GDM (n = 9)		
Variables	Mean ± SD	Mean ± SD	t-test	p-value
Weight at Screening (kg)	73.5 **±** 14.9	73.4 **±** 9.3	0.033	0.973
Height (m)	1.6 **±** 0.1	1.6 **±** 0.1	0.634	0.527
Parity	1.3 **±** 1.1	1.4 **±** 1.1	0.334	0.738
Body Mass Index (kg/m^2^)	28.6 **±** 5.5	27.9 **±** 2.4	0.323	0.747
50-g GCT result (mmol/L)	5.8 **±** 1.2	8.2 **±** 1.0	5.832	**< 0.001**
Systolic BP (mmHg)	104.8 **±** 10.6	105.5 **±** 11.3	0.199	0.842
Diastolic BP (mmHg)	65.1 **±** 9.9	68.4 **±** 11.2	0.939	0.349

2013 WHO GDM Diagnostic Criteria used to make diagnosis of GDM, GCT: Glucose Challenge Test

In the same cohort of study participants, women who had GDM when the 2013 WHO GDM diagnostic criteria was used (7.7%) were more than women diagnosed with GDM with the 1999 WHO GDM diagnostic criteria (2.6%) (Table 3). All the women diagnosed with GDM with the 2013 WHO criteria all had abnormal fasting plasma glucose (Table 4).

**Table 3:** prevalence of GDM using 1999 WHO and 2013 WHO criteria

Diagnostic criteria	GDM (N = 117)	Frequency (n)	Percentage (%)	Prevalence (%)
**2013 WHO**				
	Negative	108	92.3	7.7%
	Positive	9	7.7	
**1999 WHO**				
	Negative	114	97.4	2.6%
	Positive	3	2.6	

n - number

**Table 4:** prevalence of GDM based on a single component of the OGTT

Component of 75-g OGTT	Frequency n (%)	Prevalence (%) n/N
Fasting plasma glucose only	9 (100)	7.7
1-hour plasma glucose only	1 (11.1)	0.9
2-hour plasma glucose only	3 (33.3)	2.6

n = Total number of women with GDM (2013 WHO Criteria) N = Total number of women in the study population (117)

## Discussion

The prevalence of GDM in this study was 7.7% (using 2013 WHO criteria) is comparable with the findings of 8.3% and 8.1% reported by Anzaku *et al*. [12] in Jos and Olagbuji *et al*. [13] in Ekiti, Nigeria respectively but less than 13.9% reported by Kuti *et al*. [6] in Ibadan, Nigeria. The variation in prevalence may be related to differences in study populations, methodologies and criteria used in the diagnosis of GDM. Anzaku *et al*. and Kuti *et al*. used the 1999 WHO criteria, Olagbuji *et al*. used the 2013 WHO criteria and Kuti *et al*. studied high risk women only.

The prevalence of GDM in this study varied with the diagnostic criteria used. The use of 2013 WHO diagnostic criteria yielded a prevalence of 7.7% while 1999 WHO diagnostic criteria resulted in a GDM prevalence of 2.6% in the same study population. This supports the assertion that the prevalence of GDM is influenced by the diagnostic method and the study population [14]. The varying prevalence of GDM in this study with different diagnostic criteria used has been reported by various studies [13, 15] while some studies did not demonstrate significant change in prevalence with diagnostic criteria used [9, 16]. Olagbuji *et al*. [13] in the study in Ekiti, Nigeria reported a GDM prevalence of 8.1% with the 2013 WHO, 7.5% with the IADPSG and 3.8% with the old 1999 WHO criteria in the same population. They noted an increase in prevalence with the new 2013 WHO and IADPSG criteria when compared with the old 1999 WHO criteria as also noted in our study. A similar finding was noted in an Australian study by Moses *et al*. [15] However, Imoh *et al*. [9] in Jos, Nigeria and Zhu *et al*. [16] in China noted no significant difference in prevalence with 1999 WHO and 2013 WHO criteria.

There was no significant difference in the clinical characteristics of women without GDM and women diagnosed with GDM in our study using the 2013 WHO diagnostic criteria as seen in Table 1 and Table 2. These clinical characteristics included previous delivery of macrosomic babies, previous history of unexplained intrauterine fetal demise and positive family history of Diabetes Mellitus in first degree relatives. These findings of similar characteristics undermine the use of selective risk factor-based screening for GDM with the implementation of the 2013 WHO diagnostic criteria.

Table 4 shows the prevalence of GDM based on a single component of the OGTT result using 2013 WHO criteria. All (100%) the women with gestational diabetes were identified by the fasting plasma glucose value only, 11.1% of women with GDM were identified by 1-hour plasma glucose value only and 33.3% by 2-hour plasma glucose value only. Majority of the women diagnosed with gestational diabetes in the study were identified by the fasting plasma glucose value which is similar to findings by Olagbuji *et al*. [13] and Trujillo *et al*. [17].

## Conclusion

The prevalence of GDM varies with diagnostic criteria and use of the recent criteria is associated with 2 to 3-fold increase in its prevalence. Women with GDM had similar clinical characteristics with women without GDM. With the 2013 WHO diagnostic criteria, a minimum of fasting plasma glucose in resource poor settings can be considered to identify women with GDM.

### What is known about this topic

- The prevalence of GDM is on the rise;
- The use of the 2013 WHO GDM diagnostic criteria contributes to the rise in prevalence of GDM.

### What this study adds

- With the implementation of the 2013 WHO criteria: the prevalence of GDM rises by 2.5-3 folds in rural Nigeria; clinical risk factors may be of no use in screening women for GDM; the fasting blood component of OGTT alone identifies almost all women with GDM.
